# Computational Fluid Dynamics Modeling of Respiratory Airflow in Tracheobronchial Airways of Infant, Child, and Adult

**DOI:** 10.1155/2018/9603451

**Published:** 2018-10-31

**Authors:** Endalew Getnet Tsega

**Affiliations:** Department of Mathematics, Indian Institute of Technology Roorkee, Roorkee, India

## Abstract

During human growth and development from infancy to adulthood, dramatic changes occur in the respiratory system. It is important to understand respiratory airflow in different age groups in age-specific treatment of respiratory disorders. This study numerically investigated the age-related effects on inspiratory and expiratory airflow dynamics in four-generation lung airway models under normal breathing conditions. Tracheobronchial airway models of infant (6 month old), child (5 years old), and adult (25 years old) from sixth to ninth generations were constructed for the study. Computational fluid dynamics (CFD) was used to solve the equations governing the airflow. Results of this study indicate that as age increases, airflow velocity, pressure, and wall shear stress decrease for both inspiration and expiration in this particular subregion of the respiratory tract. During inspiration, the splitting of velocity streamlines at bifurcations increases with age. The opposite situation merging happens during expiration, and it also increases with age. The level of splitting and merging of streamlines here reflects the influence of respiratory mechanics in the age groups. The computational models provide new information on characteristics and patterns of age-dependent respiratory airflow in the sixth to ninth generations of tracheobronchial airways and can be applied in other generations.

## 1. Introduction

During growth and development from infancy to adulthood, there is a change in lung volume, airway size, alveoli size and number, shape and stiffness of the thorax, and respiratory muscle strength [1–3]. The human respiratory system alters in response to these changes. Understanding the respiratory airflow dynamics in airways of different age groups is very useful to ensure appropriate medical treatment. It is very difficult to understand the flow characteristics in human airways by measuring experimentally because of the complex structure of the airways. With the advent of high-speed digital computers, CFD is widely used by researchers and medical scientists in modeling and simulation of the airflow dynamics in human airway models. The CFD models have been used for understanding the airflow characteristics, assessing effects of medical treatments, and optimizing drug delivery in the human respiratory tract [4].

To model the airflow dynamics in the airways of different age groups using CFD, detailed information on changes of anatomical and physiological parameters during growth is required. Some researchers investigated such changes with age. Hofmann [5] developed a mathematical model to describe age-dependent alterations of dimensions of airways and respiratory parameters (tidal volume and respiratory rate). In normal breathing, the respiratory rate decreases with age as tidal volume and minute ventilation increase [5, 6]. Isaacs and Martonen [7] presented an age-dependent model of tracheobronchial airways using the symmetric Weibel model as a baseline. Dunnill [8] conducted a quantitative study of postnatal lung growth. He concluded that the increase in the number of alveoli mainly occurs in the first eight years.

Numerous studies have been carried out to investigate the airflow characteristics and particle transport and deposition in bifurcating airways of different age groups. Deng et al. [9] investigated particle deposition in infant, child, and adult bifurcating airways by using CFD and showed the age effects in the deposition. They selected two regions, the upper airways G3–G6 and the lower airways G9–G12 of the symmetric Weibel model in the simulations. Oakes et al. [10] performed numerical simulations of airflow in subject-specific infant, child, and adult pulmonary conducting airways. They used 3D airway models based on CT scan images. They assessed the influence of airway geometry and respiratory mechanics evolution on inspiratory and expiratory airflow dynamics and showed fundamental differences of the airflow characteristics among the age groups. Liu et al. [11] discussed inspiration and expiration airflow pattern and particle deposition in children's upper respiratory tract model.

The objective of this study was to investigate quantitatively the inspiratory and expiratory airflow characteristics (velocity, pressure, and wall shear stress) in tracheobronchial airways (G6–G9) of infant, child, and adult using CFD modeling. Computational results of airflow characteristics and airflow patterns are compared for the different age groups. The influence of age-dependent changes in airway geometry and respiratory parameters on airflow dynamics is assessed in this subregion of the respiratory tract.

## 2. Methods

### 2.1. Airway Models

**Figure 1:**
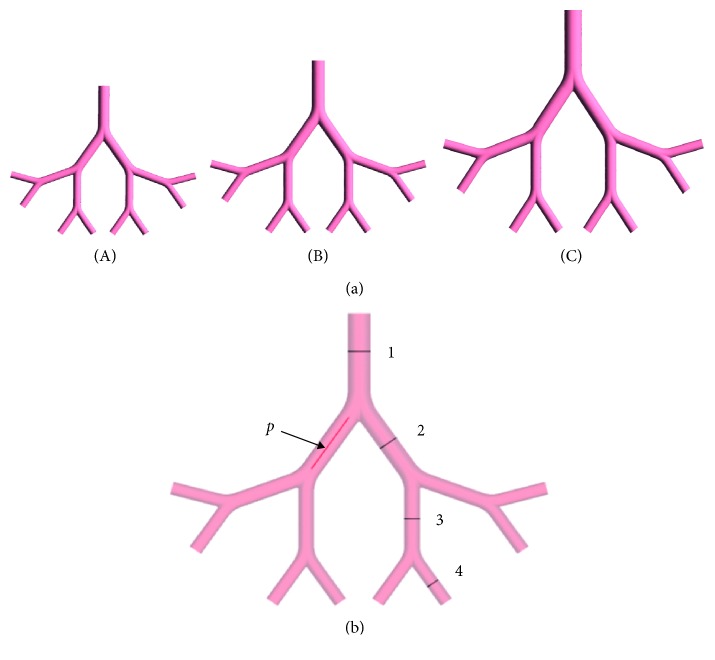
(a) Schematic view of the tracheobronchial airway model (G6–G9) of (A) infant, (B) child, and (C) adult and (b) cross sections and flow path line in an airway model.

Symmetric in-plane tracheobronchial airway (G6–G9) models of infant (6 months old), child (5 years old), and adult (25 years old) were constructed based on the Weibel 23-generation pulmonary model [12] using SOLIDWORKS, 3D CAD design software. The age-dependent dimension of the airway was adopted from [5, 7]. The bifurcation angle for each generation was 70°. The mathematical description of the morphologically realistic bifurcation model of Hegedűs et al. [13] was taken into consideration in the construction. The details of the geometric parameters are summarized in Table 1.  Figure 1 shows the schematic view of the airway models for infant, child, and adult. Note that, in Table 1, *L*, *D*, *R*, and *r* represent length, diameter, outer curvature radius, and carinal curvature radius, respectively.

### 2.2. Mesh Generation

After importing the 3D airway models in ANSYS Fluent 16.2 software, unstructured tetrahedral meshes with inflation layers were generated. A mesh independence study on the flow solution was performed by comparing the average velocity of five points on the axis of right seventh generation (on flow path line *p* (Figure 1(b))) for each age group with the inlet flow rate at the sixth generation. The normal inspiratory flow rate is taken in the inlet for each case. Meshes refining was carried out according to the grid convergence index suggested by Longest and Vinchurkar [14]. The number of cells taken in the mesh independent study was 47194, 183145, 29355, and 578851 for infant, 151031, 332297, 698154, and 886962 for child, and 427537, 917305, and 1601430 for adult. With mesh convergence tolerance <0.3%, the number of cells selected in the study was 578851, 886962, and 1601430 for infant, child, and adult, respectively.

### 2.3. Governing Equations

Air within the human respiratory tract is considered to be a homogeneous, Newtonian, and incompressible fluid. The Womersley numbers at the inlet of G6 are about 0.25, 0.34, and 0.44 for infant, child, and adult, respectively, during inspiration under normal condition. These indicate that the unsteady effects of the flow fields are relatively minor [15]. Thus, the airflow in the airway model G6–G9 is assumed to be steady. In the regions of the airway models for this study, the Reynolds number is sufficiently low so that laminar flow is assumed [16]. The governing equations for the airflow are the continuity and Navier–Stokes equations for viscous incompressible Newtonian fluid. In vector notation, these are

(1) $$\begin{array}{c} \nabla .v=0, \end{array}$$

(2) $$\begin{array}{c} \rho \left(v.\nabla v\right)=-\nabla p+\mu \nabla^{2}v, \end{array}$$

where *v* is the velocity vector, *p* is the static pressure, *ρ* is the density, and *μ* is the dynamic viscosity of the air. Taking U.S. Standard Atmosphere Air Properties Data at the sea level, the density and dynamic viscosity of air are taken to be *ρ*=1.225  kg/m^3^ and *μ*=1.7894 × 10^−5^  kg/(ms).

### 2.4. Boundary Conditions

The velocity inlet and pressure outlet conditions were used at the inlets and outlets of the airway models, respectively, while the no-slip condition was imposed on the walls for inspiratory and expiratory airflow modeling. In this study, the breathing parameters tidal volume and respiratory rate were obtained from [5] and the inspiration-to-expiration time ratio (I : E) was adopted from [17] (Table 2). The Weibel model for flows in lung airways was used to obtain the corresponding flow rates and the inlet velocities.

### 2.5. Numerical Methods

The finite-volume-based CFD software ANSYS Fluent 16.2 was used for modeling of airflow in the constructed airway models. The governing equations were solved using a pressure-based solver. The SIMPLE algorithm was applied in the CFD solver for the pressure-velocity coupling. The second-order discretization scheme was used for the pressure term, and the second-order upwind discretization scheme for momentum terms. The underrelaxation factors 0.3 and 0.5 are selected for pressure and momentum, respectively. A residual of <10^−5^ was taken for the convergence criteria.

## 3. Computational Validation

**Figure 2:**
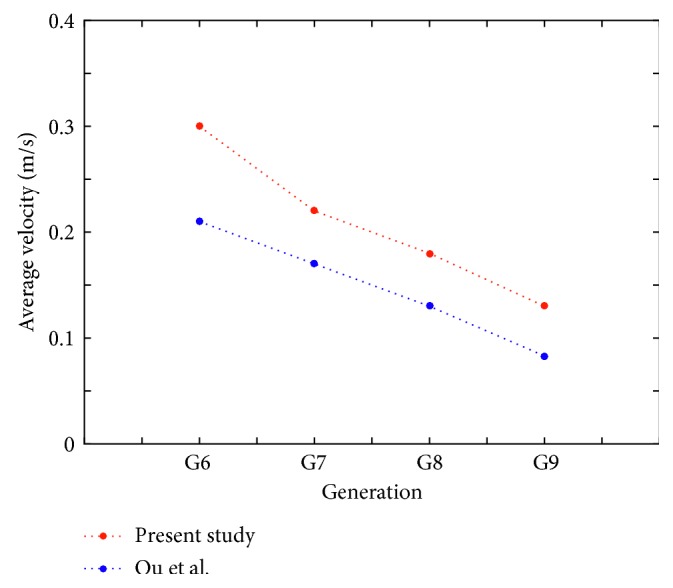
Comparison of average velocities in middle cross sections of generations of the adult airway model.

The geometric modeling method of human airways used in this study is similar to that of Deng et al. [9] and Chen et al. [18]. The CFD modeling of the inspiratory airflow in the adult human airways model was validated. The average velocities in the middle cross sections (Figure 1(b)) of G6–G9 generations of the airway model were computed with the inspiratory flow rate 4.8 L/min. The computed results were compared with the results reported by Ou et al. [19] for the same generations and inspiratory flow rate. Comparison of the average velocities is displayed in Figure 2. The difference in magnitude might result from variations of the airway models (in-plane and off-plane), airway dimensions, and the adopted boundary conditions.

## 4. Results and Discussion

Computational results of inspiratory and expiratory airflow in the models of tracheobronchial airways (G6–G9) of infant, child, and adult are presented in this section. The airflow characteristics such as velocity, pressure, and wall shear stress are selected for discussion and comparison. To make clear visibility, the figures displayed in this section are not scaled according to the dimensions of the airway they represent. All colour plots of a flow characteristic are shown along the same scale for inspiration and expiration.

### 4.1. Velocity Distribution

**Figure 3:**
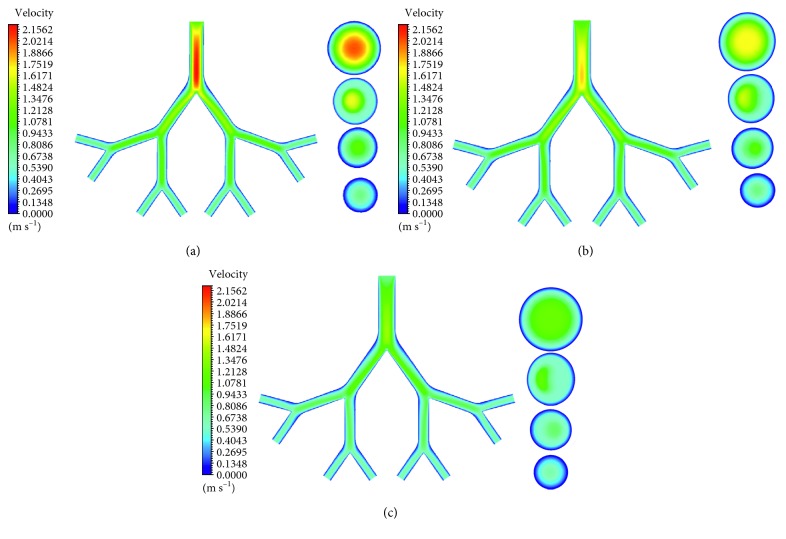
Velocity contours at midplane and middle cross sections (1–4) of G6–G9 generation of (a) infant, (b) child, and (c) adult airway models during inspiration.

**Figure 4:**
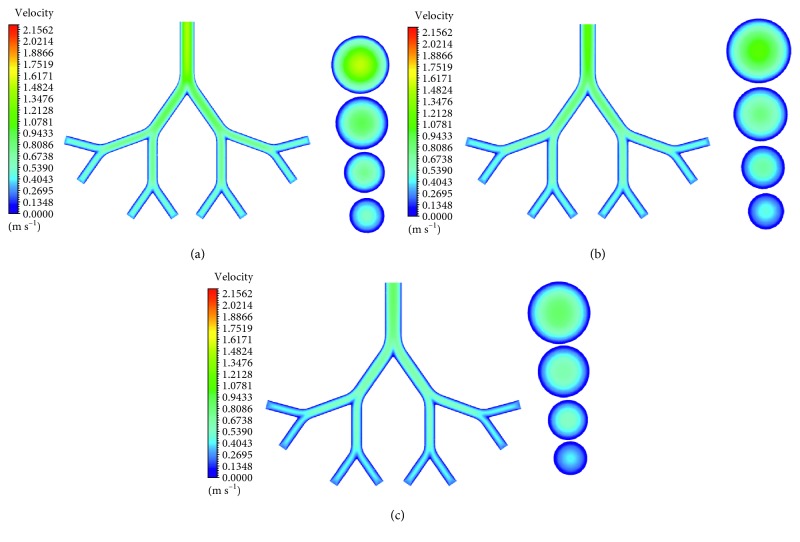
Velocity contours at midplane and middle cross sections (1–4) of G6–G9 generations of (a) infant, (b) child, (c) and adult airway models during expiration.

Figures 3(a)–3(c) show velocity contours at midplane (*z* = 0) and middle cross sections (1–4) of the G6–G9 generations during inspiration in the infant, child, and adult airway models. Analogously, velocity contours during expiration are displayed in Figures 4(a)–4(c). As age increases, airflow velocity decreases for both inspiration and expiration. The maximum velocities during inspiration for infant, child, and adult are 2.22, 1.82, and 1.41 m/s, respectively. The corresponding velocities during expiration are 1.49, 1.12, and 0.89 m/s. For all age groups and for both respiratory cases, these maximum values occur in G6. The airflow velocities decrease down to the generations. Even though the airway models are symmetrical, the velocity distributions are not symmetrical in the airways.

**Figure 5:**
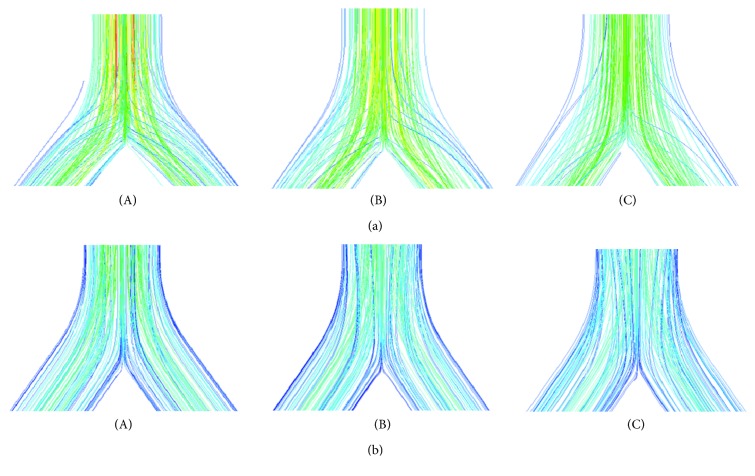
Streamlines at the first bifurcation of (A) infant, (B) child, and (C) adult airway models during (a) inspiration and (b) expiration.

The airflow patterns during inspiration and expiration for different age groups are presented using velocity streamlines. The splitting of velocity streamlines at all bifurcations increases with age during inspiration as illustrated in Figure 5 for the first bifurcations. The opposite situation merging happens during expiration, and it also increases with age. The level of splitting and merging of streamlines here reflects the influence of respiratory mechanics in the age groups.

**Figure 6:**
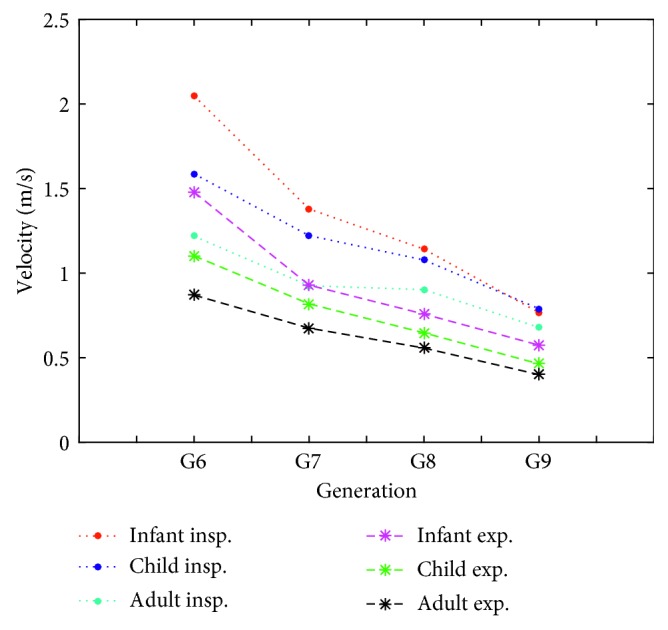
Comparison of inspiratory and expiratory airflow velocities at the center of middle cross sections of G6–G9 generations for different age groups airway models.

Figure 6 compares the velocities at the centers of the middle cross sections of G6–G9 generations for infant, child, and adult airway models during inspiration and expiration. At the same generations of the airway models, the differences of the velocity values among the age groups decrease down to the generations during inspiration and expiration.

### 4.2. Pressure Distribution

**Figure 7:**
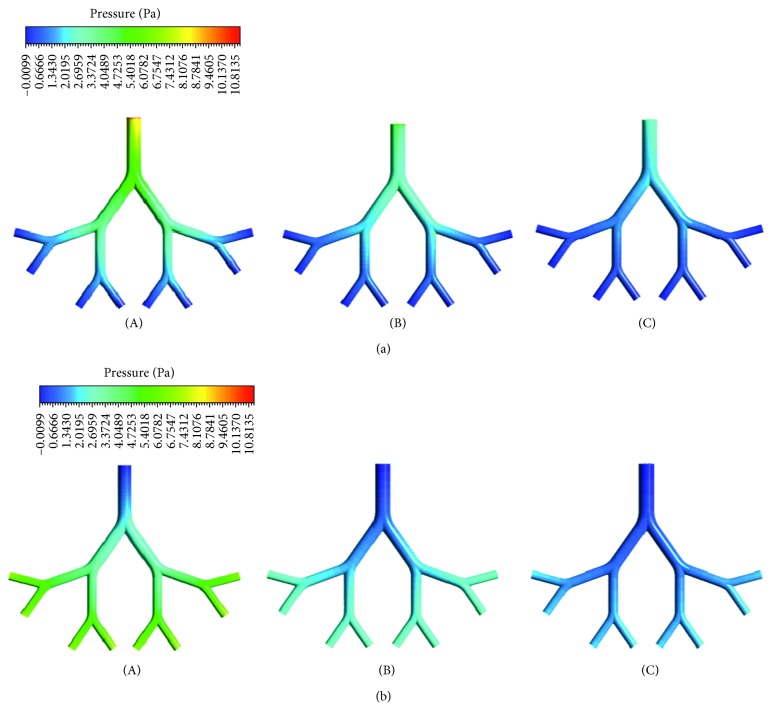
Wall pressure distribution in the airway model of (A) infant, (B) child, and (C) adult during (a) inspiration and (b) expiration.

**Figure 8:**
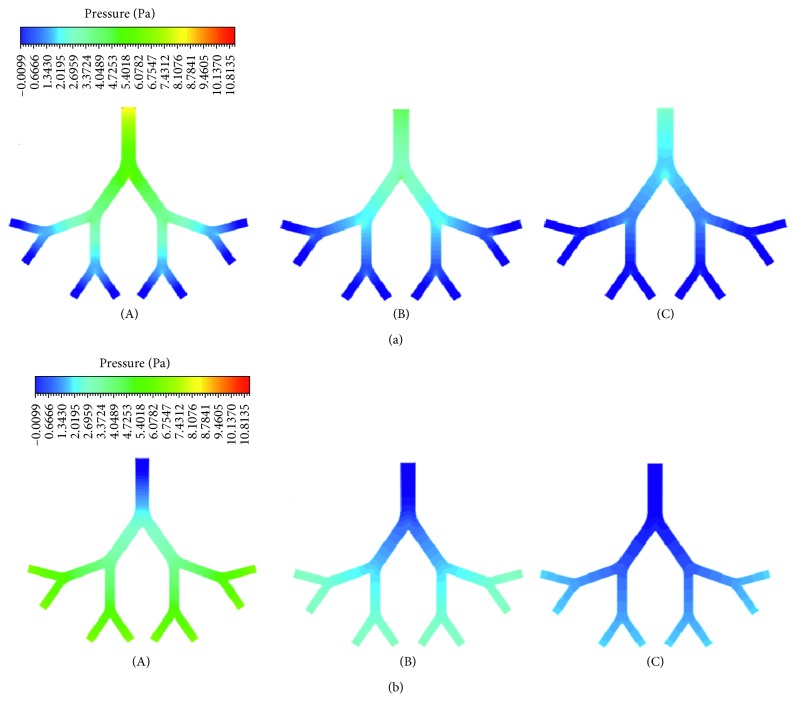
Pressure contours at midplane of (A) infant, (B) child, and (C) adult airway models during (a) inspiration and (b) expiration.

Figure 7 illustrates the computational results of wall pressure contours in the infant, child, and adult airway models during inspiration and expiration. The pressure in the airway models increases with age. It is higher for inspiration than expiration. The pressure drops on the infant, child, and airway models are, respectively, 11.15, 6.54, and 3.71 Pa during inspiration and 7.11, 3.45, and 1.99 Pa during expiration. The wall pressure decreases down to the generations during inspiration and increases up to generations during expiration. This reveals the fact that air flows from a region of higher pressure to a region of lower pressure in the respiratory tract. The wall pressure is highest at the entrance of G6 for each age group during inspiration.  Figure 8 shows the pressure distribution in midplane of the airway models for the age groups during inspiration and expiration.

**Figure 9:**
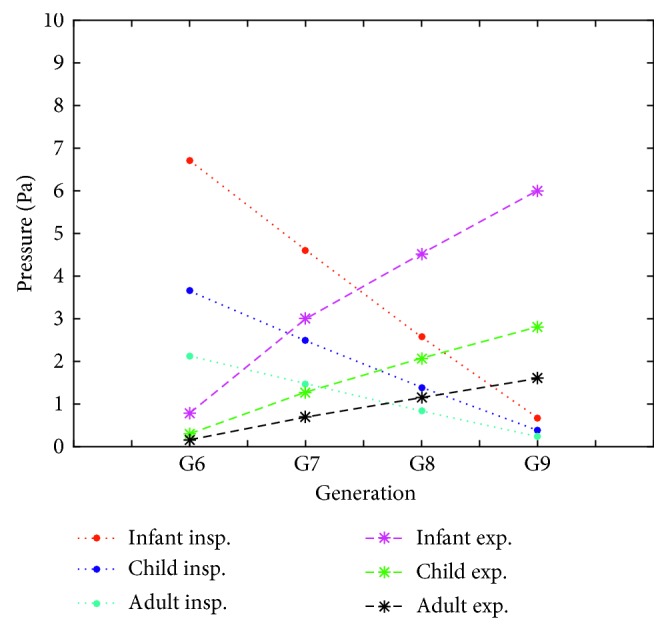
Comparison of inspiratory and expiratory airflow pressure drop at the center of middle cross sections of G6–G9 generations for different age group airway models.

Figure 9 compares the pressure drops among the age groups during inspiration and expiration at the center of the middle cross section of the airway generations. The difference of pressure drops at the same generation between infant and child is greater than that between child and adult during inspiration and expiration.

### 4.3. Wall Shear Stress

**Figure 10:**
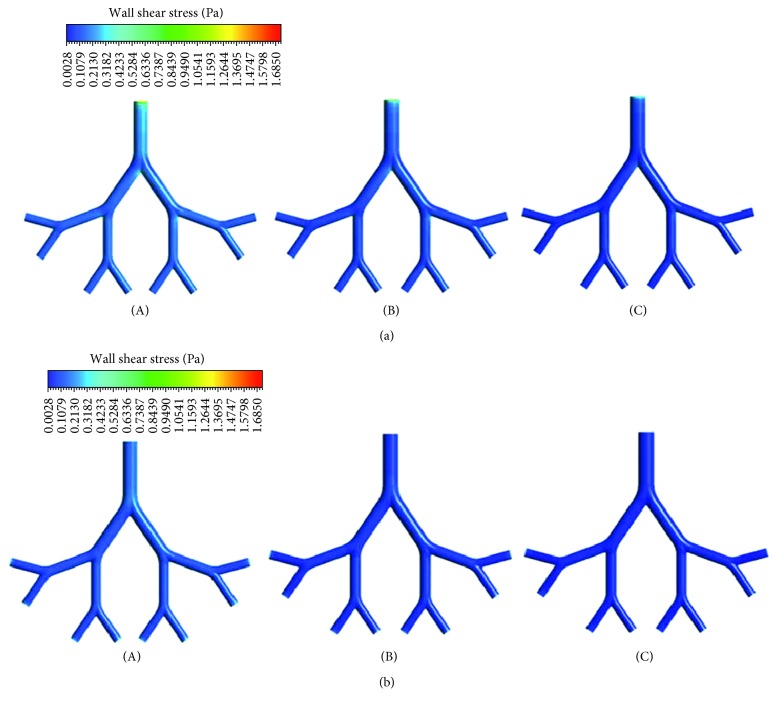
Wall shear stress distribution in the airway models of (A) infant, (B) child, and (C) adult during (a) inspiration and (b) expiration.

The wall shear stress contours in the airway models of different age groups for inspiration and expiration are presented in Figure 10. The variations of wall shear stress are similar to those of the airflow velocities in the airway models. The wall shear stress values are small and vary little with airway generations. The maximum wall shear stress values for infant, child, and adult are, respectively, 1.74, 1.23, and 0.66 Pa during inspiration and 0.44, 0.28, and 0.16 Pa during expiration. The higher value of wall shear stress in the infant airway model indicates that infants are more sensitive to the airway wall damages than children and adults.

## 5. Conclusions

The study showed that respiratory airflow dynamics in the airway generations G6–G9 of infant, child, and adult is different; the airflow characteristics (velocity, pressure, and wall shear stress) decrease with age during inspiration and expiration; and there is variation of airflow pattern among the three age groups and between the two phases of respiration. The influences of the respiratory mechanics on the airflow in the age groups were reflected in the distribution of the velocity streamlines in the CFD modeling. The study further showed that wall shear stress is very small in the airways of the age groups and relatively higher for infants. This supported evidence that infants are more sensitive to the damages in the airway walls.

## Figures and Tables

**Table 1:** The geometric dimensions of the airway models.

Generation	*L* (mm)			*D* (mm)			*R* (mm)			*r* (mm)		
	Infant	Child	Adult	Infant	Child	Adult	Infant	Child	Adult	Infant	Child	Adult
6	3.5	5.6	8.8	1.0	1.8	2.8	1.8	3.0	4.6	0.09	0.15	0.23
7	2.9	4.7	7.4	0.9	1.5	2.3	1.4	2.4	3.6	0.07	0.12	0.18
8	2.5	4.0	6.3	0.7	1.2	1.8	1.2	2.0	3.0	0.06	0.10	0.15
9	2.1	3.3	5.3	0.6	1.0	1.5	—	—	—	—	—	—

**Table 2:** Respiratory parameters used in the CFD modeling.

Age group	Tidal volume (mL)	Respiratory rate (breaths/min)	Minute ventilation (mL/min)	Inspiration-to-expiration time ratio	Inspiratory flow rate (L/min)	Expiratory flow rate (L/min)
Infant	39	36	1404	1 : 1.5	3.51	2.34
Child	181	20	3620	1 : 1.7	9.77	5.75
Adult	500	14	7000	1 : 1.7	18.90	11.12

## Data Availability

There is no data used in the research.
